# Andexanet alfa for the reversal of anticoagulation: Dutch practice data

**DOI:** 10.1016/j.rpth.2026.103438

**Published:** 2026-04-01

**Authors:** Jurjen F. Krommenhoek, Wendy Beekman, Flip Kor, Pim Gal, Willemijn E.M. Berkhout, Karina Meijer, Dionne C.W. Braeken, Frederikus A. Klok, Hugo ten Cate, Renske H. Olie

**Affiliations:** 1 Department of Medicine – Thrombosis and Hemostasis, Leiden University Medical Center, Leiden, The Netherlands; 2 Medical Department, AstraZeneca B.V., The Hague, The Netherlands; 3 Department Real World Evidence, LOGEX, Amsterdam, The Netherlands; 4 Department of Hematology, University Medical Center Groningen, Groningen, The Netherlands; 5 Thrombosis Expertise Center, Maastricht University Medical Center+, Maastricht, The Netherlands

**Keywords:** andexanet alfa, anticoagulants, factor Xa inhibitors, hemorrhage, vitamin K antagonists

## Abstract

**Background:**

Andexanet alfa is a reversal agent for factor Xa (FXa) inhibitors (rivaroxaban and apixaban) and is available for the treatment of severe FXa inhibitor-associated bleeding. However, an increase in thrombotic events after administration has been reported.

**Objectives:**

This multicenter Dutch observational study aimed to provide insights into the characteristics, usage patterns, and clinical outcomes of the Dutch patient population treated with andexanet alfa.

**Methods:**

We included all patients treated with on- and off-label andexanet alfa from 6 of the 11 hospitals in the Netherlands that prescribed andexanet alfa between June 2019 and December 2023. Data were collected by LOGEX, a Dutch healthcare data company, using healthcare administrative data and questionnaires completed by clinicians at participating centers. Patient characteristics, details related to andexanet alfa administration, and 30-day clinical outcomes, including thrombotic events and all-cause mortality, were collected.

**Results:**

A total of 217 patients received andexanet alfa, including 192 treated on-label and 25 off-label. In the on-label group, the median age was 77 years (IQR, 69-82 years), and intracranial hemorrhage was the most common indication for reversal (61%). Most patients received a low dose (≤1000 mg) and were administered andexanet alfa within 4 hours of hospital admission (79%). The overall 30-day cumulative incidence of thrombotic events was 4.6%, and no thrombotic events occurred after anticoagulation was reinitiated. All-cause mortality was 34%, with similar rates between the on- and off-label groups.

**Conclusion:**

Andexanet alfa was primarily prescribed for the reversal of FXa inhibitors in patients with intracerebral hemorrhage. We observed a lower incidence of thrombotic events than reported in clinical trials.

## Essentials

• Andexanet alfa was primarily administered to reverse factor FXa inhibitors in patients with intracerebral hemorrhage.
• Few patients received andexanet alfa at a high dose or off-label.
• A lower incidence of thrombotic events was observed than that reported in previous clinical trials.
• A high rate of all-cause mortality was observed, reflecting an unselected cohort of patients treated with andexanet alfa.

## 1 Introduction

The widespread adoption of direct oral anticoagulants, particularly factor Xa (FXa) inhibitors (eg, apixaban, rivaroxaban and edoxaban), has led to a clinical need for safe and effective reversal strategies in cases of life-threatening or uncontrolled bleeding. Prothrombin complex concentrates (PCCs) have been recommended by guidelines and used off-label as prohemostatic agents for FXa inhibitors since the introduction of direct oral anticoagulants into daily practice [1,2]. Formal high-quality studies investigating the safety and efficacy of PCCs in this setting are lacking [3], with available evidence primarily derived from single-arm observational studies or studies in healthy volunteers [[4], [5], [6], [7], [8]].

At the time of this study, andexanet alfa was approved in 2018 for clinical use in the United States (US) and in 2019 in Europe for the reversal of FXa inhibitors in cases of life-threatening or uncontrolled bleeding. However, the US Food and Drug Administration issued a safety warning in December 2025 due to an elevated rate of thrombotic events. Subsequently, the manufacturer voluntarily withdrew it from the US market [9]. By contrast, andexanet alfa remains approved and marketed in Europe.

The original approval was based on the results of the The Andexanet Alfa, a Novel Antidote to the Anticoagulation Effects of Factor Xa Inhibitors (ANNEXA-4) trial, a single-arm study demonstrating that reversal of FXa inhibitors with andexanet alfa resulted in good to excellent hemostasis in 82% of patients [10,11]. These findings were later confirmed by the ANNEXA-I study, which found that the standard of care was site- and physician-dependent (85% PCC use); andexanet alfa reduced intracranial hematoma expansion but did not improve survival or functional outcomes [12].

In both studies, initial concerns were raised about the safety of andexanet alfa, as patients treated with andexanet alfa experienced a higher rate of thrombotic events [10,12]. In the ANNEXA-4 trial, 34 patients (10%) experienced thrombotic events during follow-up (vs 5.3% in the standard-of-care group). Of these, 26 occurred prior to the resumption of anticoagulation. The ANNEXA-I trial also reported a similar rate of thrombotic events; however, it did not provide specific information on the relationship between these events and the timing of anticoagulation resumption.

Since approval, andexanet alfa has been adopted in many countries, although to varying degrees. Given the differences between a (relatively) controlled trial environment and everyday clinical practice, there is a need for data on its usage, including off-label use, dosing, and overall safety profile, including mortality and thrombotic complications. To address this gap, we conducted a nationwide observational study to describe the use, treatment patterns, and clinical care outcomes associated with andexanet alfa.

## 2 Methods

### 2.1 Study design and participants

A retrospective, multicenter cohort study was conducted across 6 Dutch hospitals. Data were extracted from electronic healthcare records for the period from June 1, 2019, to December 1, 2023. Eleven hospitals were contacted for participation, and 5 declined, primarily due to the small number of patients treated with andexanet alfa. Six hospitals participated: 3 academic and 3 nonacademic. In accordance with Dutch regulations, this retrospective study did not require patient-informed consent or formal approval from a medical ethics committee. Nevertheless, the study protocol was submitted to the Dutch Clinical Research Foundation and received approval from a non-Medical Scientific Research in People Act advisory committee prior to data analysis. The study was registered at ClinicalTrials.gov (NCT05898412).

Patients were eligible for inclusion if they met all of the following criteria:

• Age ≥18 years at the time of treatment
• Received at least 1 dose of andexanet alfa
• Did not opt out of electronic medical record data use

There were no exclusion criteria. On-label use was defined according to the current (Dutch) registered indication of andexanet alfa: reversal of FXa inhibitors (rivaroxaban and apixaban) in case of uncontrolled or life-threatening bleeding [13].

Administration was considered off-label in patients receiving edoxaban prior to acute surgery or in those who received concomitant hemostatic agents.

Tranexamic acid, PCCs, FEIBA (FVIII bypassing activity inhibitor; Takeda Manufacturing Austria AG), NovoSeven RT (activated factor VII [recombinant]; Novo Nordisk A/S), and plasma were all included in our definition of hemostatic agents. In both the off- and on-label groups, traumatic bleeding refers to any bleeding caused by trauma, including but not limited to falls, accidents, and other types of physical injury.

### 2.2 Data sources and collection

Data were collected by LOGEX Healthcare Analytics from a national administrative healthcare database. This was further augmented by manual chart review at participating centers, which captured clinical details otherwise unavailable.

### 2.3 Baseline characteristics

Demographic and medical history data were collected at the time of andexanet alfa administration, including age, sex (male or female, as recorded in the medical record), medication, indication for reversal, type of anticoagulant, relevant comorbidities, and type of bleeding. Baseline variables were reported, stratified by label specification. The timing of andexanet alfa administration was defined as the registered start time of the infusion. The decision to administer a high or low dose of andexanet alfa was made by a local clinician, in accordance with Dutch national guidelines. In patients receiving apixaban, a high dose was indicated if the patient had taken a high dose (>5 mg) within 8 hours of presentation. In patients receiving rivaroxaban, a high dose was indicated if a high dose (>10 mg) was taken within 8 hours of presentation.

### 2.4 Outcome

The primary objective was to describe the use of andexanet alfa, including its indication, practice patterns, and patient characteristics. Practice patterns were assessed by reviewing clinical treatment parameters, such as the timing of andexanet alfa administration, dosage, and the use of laboratory measurement of FXa activity prior to reversal. Additionally, 30-day outcomes of care were reported, including all-cause mortality, thrombotic events, and details related to hospitalization and follow-up. All-cause mortality during and after hospitalization and within 30 days was identified through automatic linkage between the patients’ electronic health records and the municipal registry in the event of death. Because of the retrospective design of the study, we were unable to assess hemostatic efficacy.

Thrombotic events were initially identified using a combination of Clinical Classification Software (CCSR; Agency for Healthcare Research and Quality [AHQR]) (Supplementary Table S1). Identified cases were subsequently manually validated by a local clinical researcher through chart review. Thrombotic events thus captured included objectively documented pulmonary embolism, deep vein thrombosis, acute myocardial infarction, and ischemic stroke. Each thrombotic event was subsequently scored as having either minimal, moderate, or severe impact or as a suspected cause of death.

Thrombotic events were assessed by local investigators who were experienced vascular internists, serving as both treating clinicians and as those responsible for managing the events. A thrombotic event was considered to have minimal impact if it had no lasting consequences for the patient and required no additional treatment. A thrombotic event was considered to have a moderate impact if it required further treatment or diagnostics, including hospitalization. A thrombotic event was scored as severe if it resulted in significant, lasting consequences for the patient (eg, disability). A thrombotic event was classified as a suspected cause of death when there was a strong suspicion that it may have contributed to death.

### 2.5 Statistical analysis

The distribution of demographic and clinical characteristics was summarized using descriptive statistics. For continuous variables, the mean and SD were reported when the data were normally distributed; otherwise, the median and IQR were used. Categorical variables were presented as counts and percentages. For each variable, the number of observations was reported.

A competing risk analysis was conducted to estimate the 30-day cumulative incidence of thrombotic events, with all-cause mortality as the competing event. All analyses started from the time of andexanet alfa administration. Thirty-day all-cause mortality was evaluated using the Kaplan–Meier method. As deaths were automatically recorded during this period, including after hospital discharge, all patients were administratively censored at 30 days. The cumulative incidence function was computed using the “cmprsk” [14] package in R (version 4.3.2, R Foundation for Statistical Computing).

## 3 Results

### 3.1 Demographics and patient characteristics

A total of 217 patients were included, of whom 192 were treated on-label and 25 off-label (Table 1). In the on-label group, the median age was 77 years (IQR, 69-82 years), and 59% were male (*n* = 113). The primary indication for anticoagulation was atrial fibrillation (63%), followed by venous thromboembolism (22%). Among FXa inhibitors, use was roughly balanced between apixaban (56%, *n* = 108) and rivaroxaban (44%, *n* = 84). In addition to atrial fibrillation, common comorbidities included hypertension (46%), cancer (33%), coronary artery disease (22%), and heart failure (17%). Intracranial hemorrhage (ICH) was the most prevalent indication for reversal with andexanet alfa in the on-label population (61%, *n* = 117), followed by trauma (18%, *n* = 35) and gastrointestinal bleeding (10%, *n* = 20).

**Table 1:** Baseline characteristics, bleeding episodes, and reversal treatment.

Characteristic	On-label (*n* = 192), *n* (%)	Off-label (*n* = 25), *n* (%)
Age (y), median (Q1-Q3)	77 (69-82)	73 (69-77)
Male	113 (59)	24 (96)
Comorbidities		
Hypertension	89 (46)	11 (44)
Atrial fibrillation	120 (63)	19 (76)
Heart failure	33 (17)	5 (20)
Cancer	63 (33)	9 (36)
Diabetes	35 (18)	7 (28)
Renal dysfunction	22 (12)	1 (4)
Coronary artery disease	43 (22)	10 (40)
Peripheral artery disease	14 (7)	2 (8)
Prior major bleeding	40 (21)	5 (20)
Myocardial infarction	21 (11)	5 (20)
Prior stroke	51 (27)	9 (36)
Prior venous thromboembolism	42 (22)	4 (16)
**Bleeding episode**		
Bleeding type		
ICH	117 (61)	5 (20)
Trauma	35 (18)	1 (4)
GIB	20 (10)	7 (28)
Other	20 (10)	12 (48)
Type of anticoagulant		
Apixaban	108 (56)	15 (60)
Rivaroxaban	84 (44)	8 (32)
Edoxaban		2 (8)
Indication		
Atrial fibrillation	120 (63)	19 (76)
Venous thromboembolism	42 (22)	4 (16)
Unknown or other indication	30 (15)	2 (8)
Rationale for off-label categorization		
Concomitant hemostatic^a^		18 (72)
Reversal^b^ prior to acute surgery		5 (20)
Edoxaban		2 (8)
GCS at admission		
≤8	13 (18)	1 (20)
9-12	12 (17)	2 (40)
13-15	46 (65)	2 (40)
Missing data, *n*^c^	46	0
Anti-Xa activity measured at admission	29 (15)	5 (20)
Anti-Xa activity at admission (ng/mL), median (Q1-Q3)^d^	122 (59-243)	109 (86-196)
Hemoglobin at admission (mmol/L), mean (SD)	7.8 (1.7)	6.8 (2.1)
eGFR at admission, mean (SD)	67 (20)	68 (22)
**Details of andexanet alfa administration**		
Andexanet alfa dose		
Low dose (≤1000 mg)	138 (72)	20 (80)
High dose (≥1280 mg)	54 (28)	5 (20)
Time between onset of symptoms and reversal, *n* (%)		
≤1 h	1 (1%)	1 (12.5%)
1-4 h	65 (46.1%)	5 (62.5%)
≥4 h	75 (53.2%)	2 (25%)
Missing data	*n* = 51	*n* = 17
Time between hospital admission and reversal, *n* (%)		
≤1 h	51 (34%)	3 (20%)
1-4 h	68 (45%)	4 (27%)
≥4 h	31 (21%)	8 (53%)
Missing data	*n* = 42	*n* = 10

eGFR, estimated glomerular filtration rate; GCS, Glasgow Coma Scale; GIB, gastrointestinal bleeding; ICH, intracranial hemorrhage; Q, quartile.

a Prothrombin complex concentrates, NovoSeven RT, factor VIII bypassing agent, and plasma.

b In this table, reversal refers to administration of andexanet alfa.

c As proportion of patients with ICH (*n* = 117).

d These are the levels of both rivaroxaban and apixaban, which have different therapeutic ranges. The therapeutic range for apixaban is 22 to 302 ng/mL, and for rivaroxaban, it is 9 to 361 ng/mL.

Almost all patients in the off-label group were male (96%, *n* = 24), with a median age of 73 years (IQR, 69-77 years). Off-label use was most often related to concomitant or prior administration of PCCs and/or other hemostatic agents (*n* = 18), followed by acute surgery (*n* = 5) and edoxaban use (*n* = 2). Compared with the on-label group, there was a similar distribution of comorbidities and indications for anticoagulation (Table 1). Among the off-label population, gastrointestinal bleeding was the most common indication for andexanet alfa administration.

### 3.2 Reversal

In the on-label population, patients predominantly received a low dose of andexanet alfa (≤1000 mg, 72%; Table 1). The majority of patients (79%) received andexanet alfa within 4 hours of hospital admission, and approximately half had anticoagulation reversed within 4 hours of symptom onset (46.8%). Anti-FXa activity was infrequently measured at admission (15%, *n* = 29); when assessed, the median level was 122 ng/mL (IQR, 59-243 ng/mL).

### 3.3 Thrombotic events and all-cause mortality

**Figure 1:**
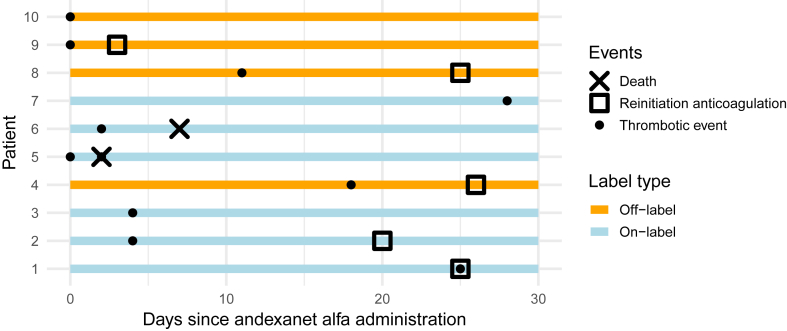
Swimmer plot of thrombotic events within 30 days following andexanet alfa administration. The swimmer plot illustrates individual patient timelines over the 30-day follow-up period after andexanet alfa administration among patients who experienced thrombotic events. Each bar represents 1 patient, with the length indicating the time from treatment to the end of follow-up (administratively censored at 30 days). Symbols denote the timing of thrombotic events (dot), death (cross), and the resumption of therapeutic anticoagulation (square), as applicable.

**Figure 2:**
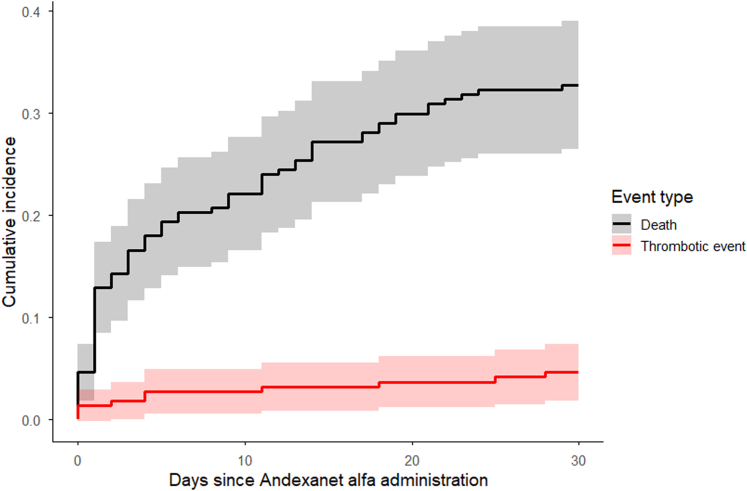
Cumulative incidence of thrombotic events during 30-day follow-up, with all-cause mortality treated as a competing event. Patients were administratively censored at 30 days.

Overall, a total of 11 thrombotic events were observed in 10 patients (Table 2 and Figure 1). The cumulative incidence of thrombotic events at 30 days was estimated at 4.6% (95% CI, 3.2%-7.6%; Figure 2) with all-cause mortality as a competing event. Among the on-label patients (*n* = 192), 6 experienced a total of 7 thrombotic events (Table 2). In contrast, among the off-label patients (*n* = 25), 4 patients each experienced 1 thrombotic event. Among all thrombotic events, 7 were of moderate severity, 1 was severe, and 3 were suspected to have directly contributed to mortality (Table 3). As shown in Figure 1, the majority of thrombotic events occurred within the first week following reversal. No thrombotic events were observed in patients who were reinitiated on anticoagulation (Figure 1).

**Table 2:** Clinical outcomes.

Variable	On-label (*n* = 192), *n* (%)	Off-label (*n* = 25), *n* (%)
Thrombotic events within 30 d	7 (3.7)	4 (16)
30-d all-cause mortality	63 (34)	9 (36)
In-hospital mortality	44 (23)	8 (32)
Discharged alive from the hospital	148 (77)	17 (68)
Surgical intervention per bleeding type		
ICH; *n*	4 (3); 117	1 (20); 5
non-ICH; *n*	24 (32); 75	4 (20); 20
Duration of hospitalization (d), median (IQR)	8 (4-15)	10 (5-21)
Proportion of patients admitted to the intensive care unit	71 (37)	5 (20)
Duration of intensive care unit stay (d), mean (SD)	1.6 (4.7)	5.9 (13)
Rehospitalization within 30 d of patients discharged alive	18 (12)	1 (6)
Anticoagulant reinitiated within 30 d	45 (23)	8 (32)
Time till reinitiation of anticoagulation (d), mean (SD)	6 (9)	3 (6)
Type of anticoagulant reinitiated		
Rivaroxaban	25 (56)	2 (25)
Apixaban	17 (38)	3 (38)
Other^a^	3 (6)	3 (38)

Percentages may not total 100% because of rounding.

ICH, intracranial hemorrhage.

a In the on-label group, 1 patient was started on dabigatran and 1 on tinzaparin (a low-molecular-weight heparin). In the off-label group, 1 patient was reinitiated on edoxaban; treatment for the other 2 patients was unknown.

**Table 3:** Characteristics and clinical impact of thrombotic events.

Patient	Index bleeding	Type of TE	On-label/off-label	Impact	Anticoagulation reinitiated at the time of TE? (yes/no)	Time since andexanet alfa administration (d)	Event description
1	ICH	PE	On-label	Moderate	No	25	Readmitted due to dyspnea. Diagnosed with PE and hospitalized for 6 d.
2	ICH	Stroke	On-label	Moderate	No	4	Initially admitted to the ICU, a brain CT scan 4 d later revealed new ischemic areas.
3	ICH	Stroke	On-label	Severe	No	4	Deterioration of neurological function observed; MRI revealed cerebral ischemia.
4	Postprocedural pericardiac bleeding	Stroke	Off-label	Moderate	No	18	Left arm paralysis, subsequently diagnosed as ischemic stroke.
5^a^	Arterial bleeding	MI	On-label	Suspected cause of death	No	0	A STEMI developed 4 h postreversal.
5^a^	Arterial bleeding	Stroke	On-label	Suspected cause of death	No	2	Sudden decrease in consciousness. CT imaging revealed recent cerebral ischemia.
6	ICH	Stroke and arterial embolism	On-label	Suspected cause of death	No	2	Ischemic cerebral infarction was identified alongside occlusion of the left limb arteries.
7	ICH	PE	On-label	Moderate	No	28	Readmitted due to pain and dyspnea. Diagnosed with PE and discharged 2 d later.
8	ICH	DVT	Off-label	Moderate	No	11	Extensive venous thrombosis of the vena cephalica occurred during treatment with low-molecular-weight heparin.
9	GIB	Peripheral arterial thrombosis	Off-label	Moderate	No	0	Occlusion of the left brachial artery due to thrombosis, treated with thrombectomy surgery.
10	GIB	MI	Off-label	Moderate	No	0	Diagnosed with cardiac ischemia, with elevated cardiac markers; conservatively treated.

Patient numbers correspond to those in the swimmer plot in Figure 1.

CT, computer tomography; DVT, deep vein thrombosis; GIB, gastrointestinal bleeding; ICH, intracranial hemorrhage; ICU, intensive care unit; MI, myocardial infarction; MRI, magnetic resonance imaging; PE, pulmonary embolism; STEMI, ST-elevation myocardial infarction; TE, thrombotic event.

a These 2 thrombotic events occurred in the same patient.

The Kaplan–Meier estimate of the 30-day cumulative incidence of all-cause mortality was 34% (95% CI, 27.0%-39.6%) in the overall cohort. Crude mortality rates were comparable between the on- and off-label populations (34% vs 36%).

### 3.4 Hospitalization and follow-up

Among the 192 patients in the on-label group, 71 (37%) were admitted to an intensive care unit or another monitored care unit, with a mean stay of 1.6 days (SD, 4.7 days). Overall, the median duration of hospitalization was 8 days (IQR, 4-15 days). Of the 148 patients discharged alive in the on-label group, 18 (12%) were rehospitalized. Of those, 2 were readmitted due to thrombotic events. In the on-label population, anticoagulation was reinitiated in 45 patients (23%), of whom 42 (96%) were restarted on FXa inhibitors. In the off-label population, 8 patients (32%) were reinitiated on anticoagulation, of whom 6 (75%) received FXa inhibitors. Upon resumption, the mean time to resumption was 6 days (SD, 9 days) in the on-label group and 3 days (SD, 6 days) in the off-label group.

## 4 Discussion

In this Dutch multicenter cohort study, we describe the use, treatment patterns, and clinical outcomes of andexanet alfa. Andexanet alfa was most commonly prescribed on-label, at a low dose, and primarily for the reversal of anticoagulation in patients with ICH. The mortality rate and incidence of predominantly arterial thrombotic events in the first 30 days were substantial. This study describes the use of andexanet alfa in routine clinical practice across all indications, including off-label use, providing a broader perspective than previous cohort studies or trials [[15], [16], [17]]. To the best of our knowledge, few reports have documented such data on off-label use [18].

Regarding clinical outcomes, we observed a high relatively all-cause mortality rate in both the on- and off-label populations. Of note, the all-cause mortality rate in our cohort was more than twice that reported in the ANNEXA-4 study [11]. It was also higher than in the ANNEXA-I study, albeit to a lesser extent (36% vs 27.8%) [12]. We offer 2 explanations for this difference. First, our study reflects routine clinical practice and includes an all-comers population. In contrast to the ANNEXA-4 trial, which excluded patients with particularly severe ICH (Glasgow Coma Scale score ≤ 7 or initial hematoma volume ≥ 60 mL), 18% of patients with ICH in our on-label cohort had a Glasgow Coma Scale score ≤ 8. In addition, our cohort had a high prevalence of cancer (33%), which was not specifically reported in the ANNEXA-4 study and is likely to be lower in that study [10]. Second, in some hospitals, andexanet alfa is reserved for cases with particularly severe bleeding as a last resort due to its cost. Our mortality rate is comparable with that reported in 2 recent registries [19,20], although both studies had longer follow-up durations.

We found a lower incidence of thrombotic events than was reported in previous studies [10,12,17,[19], [20], [21]], with rates ranging from 8% to 15%. One possible explanation for the lower incidence observed is the high mortality rate in our cohort, which may have limited the time at risk of developing thrombotic events. However, our competing risk analysis demonstrated an incidence of thrombotic events comparable to the crude incidence rate. Moreover, patients with poor prognoses may have been transitioned to palliative care programs. Beyond this point, further diagnostic investigations are often no longer pursued, which may also have resulted in missed thrombotic events.

Our study has several strengths. We report on the clinical practice of nearly all patients treated with andexanet alfa across multiple centers nationwide. This limited the risk of selection bias and enabled us to report on the off-label population, which had previously been underreported. Patients with healthcare codes indicating thrombotic events were subsequently manually reviewed to describe the events in detail, particularly their timing and severity. However, there are some limitations that warrant discussion. Thrombotic events and other clinical data may have been missed due to misclassification, as they were initially identified using healthcare codes. Although thrombotic events were subsequently verified through manual review, which improved specificity, the sensitivity of the healthcare codes used to identify thrombotic events was not formally tested. Furthermore, aside from mortality, which was automatically recorded, some patients may have been lost to follow-up after discharge, and this may not have been accounted for in our analysis. The observed rates of thrombotic events may be underestimated due to missed cases in patients who were discharged and either died out of hospital as a result of a thrombotic event or received treatment and diagnosis at a different facility.

We were also unable to capture the timing of the last FXa dose in the majority of patients, limiting our study's ability to evaluate the appropriateness of andexanet alfa dosing.

## 5 Conclusion

In our study, we report on the use of andexanet alfa and on clinical outcomes, including mortality and thrombotic events. In the Netherlands, andexanet alfa is primarily used to reverse anticoagulation in patients with ICH. We observed a relatively lower incidence of thrombotic events than in published randomized controlled trials but a higher mortality rate, which illustrates the poor prognosis of this patient population and highlights the complex balance between efficacy and safety in clinical practice.
