# Supplementary material for: Andexanet alfa for the reversal of anticoagulation: Dutch practice data
Source: Res Pract Thromb Haemost. 2026 Apr 1;10(3):103438. doi: 10.1016/j.rpth.2026.103438 (PMC13138149; doi:10.1016/j.rpth.2026.103438)
Supplement: Supplementary Table [file mmc1.docx]

**Supplementary material**
Clinical Classifications Software (CCS) group 5 were used to group related ICD-10 codes into broader categories for analysis. For example, CCS code 100 consolidates all codes for acute myocardial infarction, while CCS code 118 covers conditions like deep vein thrombosis and pulmonary embolism.

The following CCS codes were used to capture thrombotic events.

| **Supplementary table S1. CCS codes used to capture thrombotic events** | |
| --- | --- |
| CCS Code 116 | Aortic and Peripheral Arterial Embolism; Arterial Systemic Embolism or Thrombosis |
| CCS Code 118 | Phlebitis; Deep Vein Thrombosis; Thrombophlebitis and Thromboembolism |
| CCS Code 100 | Acute Myocardial Infarction |
| CCS Code 103 | Pulmonary Heart Disease; Pulmonary Embolism |
| CCS Code 112 | Transient Ischemic Attack |

For further information we refer to the Agency for Healthcare Research and Quality (AHRQ).(1)

**References**(1) AHRQ. Clinical Classifications Software (CCS) for ICD-9-CM. [Internet]. Available from: https://www.hcup-us.ahrq.gov/toolssoftware/ccs/ccs.jsp
